# The Tyrolean Founder *MLH1* Variant c.836T>G Causes Lynch Syndrome Due to a Leaky Splice Effect

**DOI:** 10.3390/biom16081200

**Published:** 2026-08-17

**Authors:** Sukanya Horpaopan, Esther Schamschula, Heidelinde Fiegl, Hannes Dapoz, Christina Lutz-Nicoladoni, Simon Schnaiter, Albert Amberger, Ulrich Strasser, Renate Lunzer, Andreas von der Heidt, Katalin Csanaky, Johannes Zschocke, Katharina Wimmer

**Affiliations:** 1 Institute of Human Genetics, Medical University Innsbruck, 6020 Innsbruck, Austria; 2 Department of Anatomy, Faculty of Medicine, Chiang Mai University, Chiang Mai 50200, Thailand; 3 Department of Obstetrics and Gynecology, Medical University Innsbruck, 6020 Innsbruck, Austria; 4 Tyrolpath Obrist Brunhuber GmbH, 6511 Zams, Austria

**Keywords:** Lynch syndrome, *MLH1*, variant classification, leaky splice effect, founder mutation

## Abstract

The identification of a pathogenic variant (PV) in one of the mismatch repair (MMR) genes confirms the diagnosis of Lynch syndrome (LS). Hence, the correct classification of MMR gene variants is of utmost importance for appropriate counselling, surveillance, and treatment of LS patients and their families. In 7/200 unrelated Tyrolean-suspected LS patients, we identified the rare variant *MLH1*:c.836T>G. Clinical and tumor data strongly indicate that this founder variant is associated with an increased risk for early-onset LS-associated tumors. We also demonstrate that the variant leads to aberrant mRNA splicing. However, the splice effect’s leakiness together with the small effect of the amino acid change p.(Val297Gly) encoded by the residual full-length transcripts in a functional assay preclude its formal classification as (likely) PV according to internationally accepted variant interpretation guidelines. The family histories of the carriers suggest that the obstacles to classify the variant as (likely) PV may be related with a reduced penetrance. Nonetheless, and despite the formal classification of *MLH1*:c.836T>G as a variant of uncertain significance, we show that carriers should undergo cancer surveillance and predictive testing should be offered to relatives. This variant illustrates the need for an improved classification framework for appropriate categorization of lower-penetrance alleles.

## 1. Introduction

Lynch syndrome (LS; MIM: #609310, #614337, #120435, #613244, #614350) is one of the most frequent cancer predisposition syndromes with an estimated prevalence of 1 in 278–354 individuals [1,2]. It is caused by a heterozygous germline pathogenic variant (PV) in one of four DNA mismatch repair (MMR) genes, *MLH1* (MIM *120436), *MSH2* (MIM *609309), *MSH6* (MIM *600678), and *PMS2* (MIM *600259), or by *MSH2* silencing caused by *EPCAM* 3′-end deletions through *EPCAM-MSH2* fusion transcripts (MIM *185535). LS patients have an increased risk for mainly colorectal cancer (CRC) and endometrium cancer (EC), and about 3% of all CRC and EC cases are attributable to LS [3,4]. LS predisposes also to cancers of the small intestine, stomach, upper urothelial tract, ovaries, bile duct, pancreas, and brain. Guidelines for cancer surveillance of MMR gene PV carriers [5] consider marked gene- and gender-dependent cancer risk variability, ranging from 11% overall cancer risk in male *PMS2* PV carriers to 85% in female *MSH2* PV carriers [6,7].

Screening all CRC and EC for MMR deficiency (MMRd), the hallmark feature of LS-associated tumors [8,9], usually accompanied by expression loss of one or more MMR protein(s) and microsatellite instability (MSI) in neoplastic cells, is the most effective way to identify suspected LS patients [10]. The identification of a (likely) PV in one of the MMR genes subsequently confirms the diagnosis of LS in a suspected patient. Identification of a variant of uncertain significance (VUS), however, constitutes a clinical challenge, as these variants are non-actionable in terms of cancer surveillance and predictive testing of relatives.

International efforts mainly driven by the International Society for Gastrointestinal Hereditary Tumours (InSiGHT) and American College of Medical Genetics and Genomics Association for Molecular Pathology (ACMG/AMP) were undertaken to reduce the number of VUSs by developing guidelines and establishing expert panels for variant classification (ClinGen InSiGHT Hereditary Colorectal Cancer/Polyposis Expert Panel Specifications to the ACMG/AMP Variant Interpretation Guidelines for MMR Genes Version 2.0.0). Still, as of April 2026, 42% (*MLH1*) to 54% (*PMS2*) of the MMR gene variants deposited in the ClinVar database (https://www.ncbi.nlm.nih.gov/clinvar/ (accessed on 1 April 2026)) are classified as VUSs or have conflicting classifications. About 50% of these VUSs are deposited by a single submitter, suggesting they have not been frequently seen, and the majority are exonic variants with predicted missense effect. Often, functional studies showing an impact of the amino acid alteration on the MMR capacity of the resulting gene product will bring them into an actionable variant class. Furthermore, intronic and exonic VUSs may impact gene product function by altering mRNA splicing. Therefore, showing a splice effect by analysis of patient-derived transcripts and/or ex vivo by minigene experiments may provide strong evidence in favor of the pathogenicity of a VUS [11].

Here, we present clinical and tumor data as well as extensive functional analyses of the rare and so far unclassified variant *MLH1*:c.836T>G, detected in seven apparently unrelated suspected LS patients from Tyrol.

## 2. Materials and Methods

All aspects of this study were conducted according to the Declaration of Helsinki. This study was approved by the Ethics Committee of the Medical University Innsbruck (MUI, Reference No. 1304/2025).

### 2.1. Patients and Study Cohorts

Between April 2009 and October 2025, 706 index patients from non-related families were referred to the Institute of Human Genetics, Medical University Innsbruck (MUI), for LS testing. Of these, 200 patients had a German surname and lived in Tyrol and, hence, are likely of Tyrolean origin. These constitute the here analyzed “Tyrolean-suspected LS patient cohort” (Table 1; Supplementary Table S1). Informed consent for genetic testing was obtained from all of them.

The endometrial cancer (EC) cohort genotyped for variant *MLH1*:c.836T>G includes 239 unselected archived tumor DNA samples obtained at primary surgery between 1989 and 2015. All patients are of Caucasian ethnicity and were collected and stored in the Biobank of the Department of Obstetrics and Gynecology, MUI. The majority of patients in this cohort (*n* = 206) are likely of Tyrolean origin according to the criteria described above. The clinicopathological data of the patients in the cohort were described previously [12,13].

The CRC cohort includes DNA samples of 316 tumors analyzed and archived between 2014 and 2022 at Tyrolpath Obrist Brunhuber GmbH, Zams, Austria. Patients whose tumors are included in this cohort lived in Tyrol and had a German surname and, hence, are likely of Tyrolean origin. The tumor MMR characteristics of this cohort are provided in Supplementary Table S2.

### 2.2. Genetic Analysis of the MMR Genes in Suspected LS Patients

Until June 2014, genetic testing of MMR genes in suspected LS patients included Sanger sequencing, as described previously [14], and MLPA analysis of the gene(s) encoding the MMR protein(s) showing expression loss in tumor tissue. To generate reliable copy number (CN) results for the *PMS2* gene regions homologous with *PMS2CL* pseudogene sequences, *PMS2* MLPAs were performed as described previously [15]. As of June 2014, next-generation sequencing (NGS) was used for sequence and CN analyses. Until March 2023, the TruSight™ Hereditary Cancer Panel (Illumina, San Diego, CA, USA) and thereafter a custom panel (Twist Bioscience, South San Francisco, CA, USA) were sequenced on a MiSeq (Illumina). Data obtained from NGS, Sanger sequencing, and MLPA were analyzed with the SEQNEXT, SEQPATIENT, and MLPA modules of the Sequence Pilot 5.4.1 software (JSI Medical Systems GmbH, Ettenheim, Germany), respectively. For CN analysis, panelcn.MOPS [16] was additionally used.

Additionally, direct cDNA sequencing of the respective gene was performed, as described by Etzler et al. [14], in patients carrying a (suspected) splice variant to assess its effect on mRNA splicing (for more details, see also “*MLH1*:c.836T>G allele transcript analysis” below). Direct cDNA sequencing was also used to exclude/identify variants escaping genomic sequencing and CN analysis in patients with a negative gDNA test result and, hence, unexplained expression loss of one or more MMR protein(s) in their tumors.

For all detected variants, nucleotide positions were numbered in accordance with the Human Genome Variation Society (HGVS) recommendations [17]. NM_000249.4, NM_000251.3, NM_000179.3, and NM_000535.7 were used as reference transcripts for *MLH1*, *MSH2*, *MSH6*, and *PMS2*, respectively. We used Priors V2.0 (http://hci-priors.hci.utah.edu/PRIORS/BRCA/index.php?gene=MLH1 (accessed on 03 April 2026)), REVEL [18], SIFT [19], MutationTaster [20], and PolyPhen2 [21] for in silico prediction of missense effects. SpliceSiteFinder-like [22], MaxEntScan [23], NNSPLICE [24], GeneSplicer [25], and ESE-finder [26], as well as SpliceAI (https://spliceailookup.broadinstitute.org/# (accessed on 11 April 2026), were used to predict splice effects. All prediction programs are incorporated in the Alamut Visual Plus version 1.3 (SOPHiA GENETICS, Rolle, Switzerland).

### 2.3. Tumor Tissue Analyses

Tumor DNA was extracted from formalin-fixed paraffin-embedded (FFPE) tumor samples using the QIAamp DNA FFPE Tissue Kit (Qiagen, Hilden, Germany) or Reliaprep FFPE gDNA Miniprep System (Promega, Madison, WI, USA) according to the manufacturers’ recommendations. DNA concentration was determined with the Nanodrop One fluorescence spectrophotometer (Thermo Scientific, Waltham, MA, USA) and samples were stored at 4 °C.

MSI analysis was performed using a multiplex fluorescence-based PCR assay with a panel of six microsatellite markers (BAT25, BAT26, BAT40, D2S123, D5S346, and D17S250) on tumor and surrounding normal tissue in patients diagnosed with cancer before the introduction of the TrueMark™ MSI assay (Applied Biosystems, Foster City, CA, USA) analyzing a lager set of only mononucleotide markers (BAT25, BAT26, BAT40, CAT-25, NR-21, NR-22, NR-24, NR-27, Abi-16, ABI-17, ABI-19, ABI-20A, and Abi-20B). The assay was performed according to the manufacturers’ instructions. Fragment length analysis was performed on an ABI PRISM 3730 Genetic Analyzer (Applied Biosystems) or the SeqStudio^TM^ Genetic Analyzer (Applied Biosystems).

Immunohistochemical (IHC) staining of MMR proteins was performed according to standard protocols using Ventana^®^ monoclonal primary antibodies, i.e., anti-MLH1, -PMS2, -MSH2, and -MSH6 (clone M1, A16-4, G219-1129, and SP93; Roche Diagnostics, Tucson, AZ, USA). IHC results were evaluated by a pathologist.

For *MLH1* promoter hypermethylation, DNA bisulfite modification was performed using the EZ DNA Methylation-Gold Kit (Zymo Research, Orange, CA, USA) according to the manufacturer’s instructions. The bisulfite-converted DNA was amplified using a biotinylated primer set targeting the *MLH1* promoter region. The primer sequences used were MLH1_meth_Fwd and MLH1_meth_Rev_Bio (biotinylated). Pyrosequencing was subsequently performed on a PyroMark Q24 system (Qiagen) to quantify methylation status. The sequencing reaction was initiated with the sequencing primer MLH1_Bis_Seq. Data were analyzed using the PyroMark Q24 2.0.6 CpG Software Module (Qiagen). To determine the percentage of methylated reference (PMR) values, Methylight analysis was done, as described [27].

BRAF p.V600E status was determined by either allele-specific real-time PCR or targeted NGS. Real-time PCR was performed on a Rotor-Gene Q (Qiagen) using the PyroMark PCR Master Mix and EvaGreen (Biotium, Fremont, CA, USA). Allele discrimination utilized 3′ terminal nucleotide-specific primers BRAF_WT_Fwd and BRAF_V600E_Fwd together with BRAF_common_Rev (all primer sequences are listed in Supplementary Table S3). Mutation status was determined by ΔCt shift analysis and confirmed by melting curve verification. Selected samples were analyzed using AmpliSeq for Illumina Focus or Comprehensive v3 Panels (Illumina). Libraries were prepared via multiplex PCR and sequenced on an Illumina platform (MiniSeq or NextSeq 550). Sequence data were analyzed with the SEQNEXT module of the Sequence Pilot 5.4.1 software (JSI Medical Systems GmbH).

To get an indication of somatic biallelic *MLH1* inactivation, tumor DNA of patients carrying the variant *MLH1*:c.836T>G was sequenced. Sequencing libraries were prepared using the TruSight^TM^ Hereditary Cancer Panel (Illumina) or a custom panel (Twist Bioscience), according to the manufacturers’ protocols. Libraries were sequenced on a MiSeq (Illumina). The SEQNEXT module of the Sequence Pilot 5.4.1 software (JSI Medical Systems GmbH) was used for variant detection and CN analysis. CN neutral loss of heterozygosity (LOH) was deduced from variant allele frequencies (VAFs) of common heterozygous polymorphisms surrounding the *MLH1* variant c.836T>G. In addition, LOH analysis was performed by single nucleotide polymorphism (SNP) array using the Infinium Global Screening Array-24 BeadChip (Illumina) according to the manufacturer’s protocol. Data were generated with the iScan^TM^-System (Illumina) and analyzed with Genome Studio v2.0 software (Illumina).

### 2.4. Haplotype Analysis of the MLH1:c.836T>G Allele

To determine if *MLH1*:c.836T>G is a founder variant and to estimate the common haplotype size, SNPs surrounding the *MLH1* locus on chromosome 3 were genotyped in all seven patients carrying this variant using the Infinium Global Screening Array-24 BeadChip (Illumina) and analyzed with Genome Studio v2.0 software (Illumina).

The maximal common haplotype around *MLH1*:c.836T>G was deduced from unphased genotypes of the seven carriers. For age estimation of the variant (i.e., estimation of time since the most recent common ancestor), the Gamma method previously described by Gandolfo et al. [28] was used applying the Genetic Mutation Age Estimator (https://shiny.wehi.edu.au/rafehi.h/mutation-dating/ (accessed on 16 April 2026)) developed by Dr. Haloom Rafehi at the Walter and Eliza Hall Institute of Medical Research, Melbourne, Victoria, Australia.

### 2.5. MLH1:c.836T>G Allele Transcript Analysis

Total mRNA was extracted from patients’ short-term PHA-stimulated lymphocyte cultures treated with puromycin (Sigma-Aldrich, St. Louis, MO, USA) using the RNeasy Mini Kit (Qiagen). RNA was reverse transcribed using the Maxima H Minus First Strand cDNA Synthesis Kit (Thermo Scientific) and random hexamer primers, as previously described [14]. PCR was performed with GoTaq Polymerase (Promega, Madison, WI, USA) and primers MLH1_E8_Fwd and MLH1_E11_Rev (Supplementary Table S3). Reverse transcriptase (RT)-PCR products were purified from a 2% Agarose gel using the PeqGOLD Gel Extraction Kit (PEQLAB, Erlangen, Germany) following the manufacturer’s protocol and Sanger sequenced using the BigDye Terminator v1.1 Cycle Sequencing Kit (Applied Biosystems) and the ABI PRISM 3730 Genetic Analyzer (Applied Biosystems).

### 2.6. Hybrid-Minigene Experiments for MLH1:c.836T>G Allele

To evaluate the splice effect of *MLH1*:c.836T>G in vitro, minigene experiments were performed, as previously described [29]. In brief, a 524 bp sequence of wildtype and variant *MLH1* exon 10 with flanking intronic sequences was PCR amplified from the patients’ DNA using primers RTB_MLH1E10_SalI_Fwd and RTB_MLH1E10_KpnI_Rev, introducing SalI and KpnI restriction sites, respectively (Supplementary Table S3), and cloned into the RTB hybrid-minigene vector previously provided by Dr. Thomas Cooper (Baylor College of Medicine) [30,31]. HEK293 cells (human embryonic kidney), HRT-18 cells (large intestine adenocarcinoma), HEC-1-B cells (MSI-endometrial adenocarcinoma), and HEC-155 cells (primary endometrial cancer) were transiently transfected with the minigene constructs. Extraction of mRNA from transfected cells, RT-PCR, and sequencing were performed as described [29]. HEK293 cell line was originally established by Dr. Alex Van Der Eb and Dr. Frank Graham at Leiden University in the Netherlands in 1973 [32]. HRT-18 cell line was originally published in 1974 by Dr. Tompkins and colleagues [33]. HEC-1-B cell line was originally established in 1968 by Dr. Hiroyuki Kuramoto and colleagues [34]. HEC-155 cell line was originally published in 2002 by Dr. Iida and colleagues [35].

### 2.7. CIMRA Assay for MLH1 p.(Val279Gly)

To functionally characterize the repair capacity of the full-length protein including the missense variant MLH1 p.Val279Gly, the cell-free in vitro MMR activity (CIMRA) assay was performed, as described previously by the laboratory at the Leiden University Medical Center (LUMC), The Netherlands [36].

### 2.8. TaqMan Assay for MLH1:c.836T>G Allele Genotyping in Tumor Cohorts

The TaqMan SNP genotyping assay (Thermo Scientific) was used to test the *MLH1*:c.836T>G in anonymous unselected EC and CRC samples. Minor groove binding (MGB) probes for wildtype (T) and variant (G) alleles were labeled with VIC and FAM dyes, respectively. In each experiment, two heterozygous positive controls, two wildtype DNA controls, and three no-template controls were included. Allelic discrimination was performed on the ABI Prism 7500 Fast Real-Time PCR System (Applied Biosystems) using 10 ng of template DNA according to the manufacturer’s instructions and analyzed by t the 7500/7500 Fast Real-Time PCR Software v2.0.6 (Life Technologies, Foster City, CA, USA).

## 3. Results

### 3.1. Seven out of Two Hundred Tyrolean-Suspected LS Index Patients Carry the MLH1 Variant c.836T>G

Our cohort of 200 unrelated Tyrolean index patients referred for LS testing includes 64 (32%) male and 136 (68%) female patients. Here, 57 (28.5%), 66 (33%), and 64 (32%) cases developed cancer before the age of 50 years, at the age of 50–60 years, and after the age 60 years, respectively. For three cases (1.5%) the age of cancer onset is unknown, and two cases had only adenomas (1%). Eight cases without cancer (4%) were tested because of a family history strongly suggestive of LS. Of the 190 patients with cancer, 96 (50.5%) cases had intestinal tract cancers with or without non-LS-associated cancers, 52 (27.6%) had EC or ovarian cancer (OC) with or without non-LS-associated cancer, 20 (10.5%) had other LS-associated cancers, i.e., gastric, urothelial, sebaceous gland, and bile duct cancer, 12/190 (6.3%) had more than one LS-associated cancer, and 10 (5.3%) had one or more non-LS-associated cancer (Table 1; Supplementary Table S1).

Results of MSI analysis in tumor tissues were reported for 98/192 (51%) patients with tumors. Microsatellite instability (MSI) and microsatellite stability (MSS) were observed in 81 (83%) and 17 (17%) of these tumors, respectively. IHC staining of all four MMR genes was performed in 127 tumors. In 59 (46.4%) of these MLH1 and/or PMS2 and in 35 (27.6%) MSH2 and/or MSH6 expression loss was found. Intact expression of all four MMR proteins was observed in 24 (18.9%) and an atypical pattern of MMR protein expression (e.g., PMS2 and MSH6 expression loss or loss of all four MMR proteins) in nine (7.1%) tumors. The MMR protein expression status in tumor tissue is unknown or data are incomplete (e.g., lack of at least one MMR IHC result) for 65 tumors (Table 1 and Supplementary Table S1). Nine of the seventeen (53%) MSS tumors expressed all four MMR proteins, indicating MMR proficiency. Five (29%) showed loss of expression of at least one MMR protein and IHC data were not available for three (18%) MSS tumors. *MLH1* promoter methylation was performed in 20 tumors with MLH1 expression loss. Three of the twenty (15%) MLH1-deficient tumors exhibited *MLH1* promoter hypermethylation. All except five MSI tumors had loss of expression of at least one MMR protein (Supplementary Table S1).

Germline testing revealed a (L)PV in 57 (28.5%) of the 200 Tyrolean-suspected LS patients. VUSs were detected in seven cases (3.5%), one of which had also a (L)PV. No potentially disease-causing MMR gene germline variant was detected in 132 cases (66%), of which four (2%) had cancers with confirmed *MLH1* promotor hypermethylation, suggesting sporadic tumors (Supplementary Table S1). In seven patients (3.5%) the variant *MLH1*:c.836T>G was found. Six of these patients had intestinal tract cancers with MSI and expression loss of MLH1 and/or PMS2 (exemplary IHC stains are shown in Supplementary Figures S1 and S2), as well as absence of *MLH1* promoter hypermethylation and BRAF V600E (tested only for one patient) at the median age at diagnosis of 52.5 years (35–74 years). One patient had MSS EC and urothelial cancer, both showing loss of MSH6 expression (Supplementary Figure S3). In this latter patient we also identified the PV *MSH6*:c.3103C>T, p.(Arg1005*) (Table 2).

*MLH1*:c.836T>G is neither reported in the population database Genome Aggregation Databases (gnomAD) nor in the InSiGHT Variant Database, the Human Gene Mutation Database (HGMD), the Catalogue of Somatic Mutation in Cancer (COSMIC), the Universal Mutation Database (UMD), and Leiden Open Variation Database (LOVD). In ClinVar it is deposited five times with different classifications. One entry classifies the variant as likely benign mainly following the classification of Tsai et al. [37], three entries interpreted this variant as VUS because of the absence of supporting evidence toward pathogenicity, and one as likely pathogenic based on lab-internal RNA data.

### 3.2. Common Haplotype Supports a “Tyrolean” Founder Effect of MLH1 Variant c.836T>G

The high frequency of *MLH1* variant c.836T>G in the Tyrolean-suspected LS patients (7/200) when compared to its frequency in public databases suggests a founder effect in the Tyrolean population. To confirm this assumption, we analyzed by SNP array whether carriers of the variant share a common haplotype. Genotyping all seven patients for SNPs on chromosome 3 revealed a common haplotype of maximal 1.1 Mb (Table 3) that was confirmed also by the analysis of a non-carrier and a carrier relative of P2. This finding substantiates the suspected founder effect. From the size of this common haplotype, ~0.88 cM (Supplementary Table S4), and assuming an independent genealogy, we estimated the age of this founder variant to be 186 generations (95% CI: 109.4–317.7 generations; Supplementary Table S5) using a method previously described by Gandolfo and colleagues that can be reliably applied to small samples [28]. Assuming an average of 25 years per generation, this equals 4650 years (95% CI: 2725–7950 years; Supplementary Table S6).

### 3.3. Tumor Analyses Indicate Somatic Inactivation of the Wildtype MLH1 Allele

The CRC characteristics, i.e., MLH1 deficiency without *MLH1* promoter methylation, already of the first patient of our cohort (P1) strongly indicated that *MLH1*:c.836T>G is associated with an increased risk for LS-associated cancers. As somatic inactivation of the wildtype *MLH1* allele would render additional evidence for the variant’s pathogenicity, we performed Sanger sequencing and later NGS and SNP array analysis of this patient’s CRC. This revealed LOH with CN loss at the *MLH1* locus and over-representation of the germline variant c.836T>G, which was found with a variant allele frequency (VAF) of 73% in the tumor (Supplementary Figure S4). These data provide strong evidence for somatic inactivation of the wildtype allele. In the CRC of patient P7, the germline *MLH1* variant c.836T>G was present with a VAF of 48% and one somatic *MLH1* PV, c.1191_1192delinsTT, p.(Gln398*), with a VAF of 24%. Sequencing and SNP array data of the MLH1-deficient tumors of the four remaining patients were not evaluable due to low DNA quality and/or low tumor cell content (Table 2).

### 3.4. MLH1 Variant c.836T>G Causes a Leaky Splice Effect

**Figure 1:**
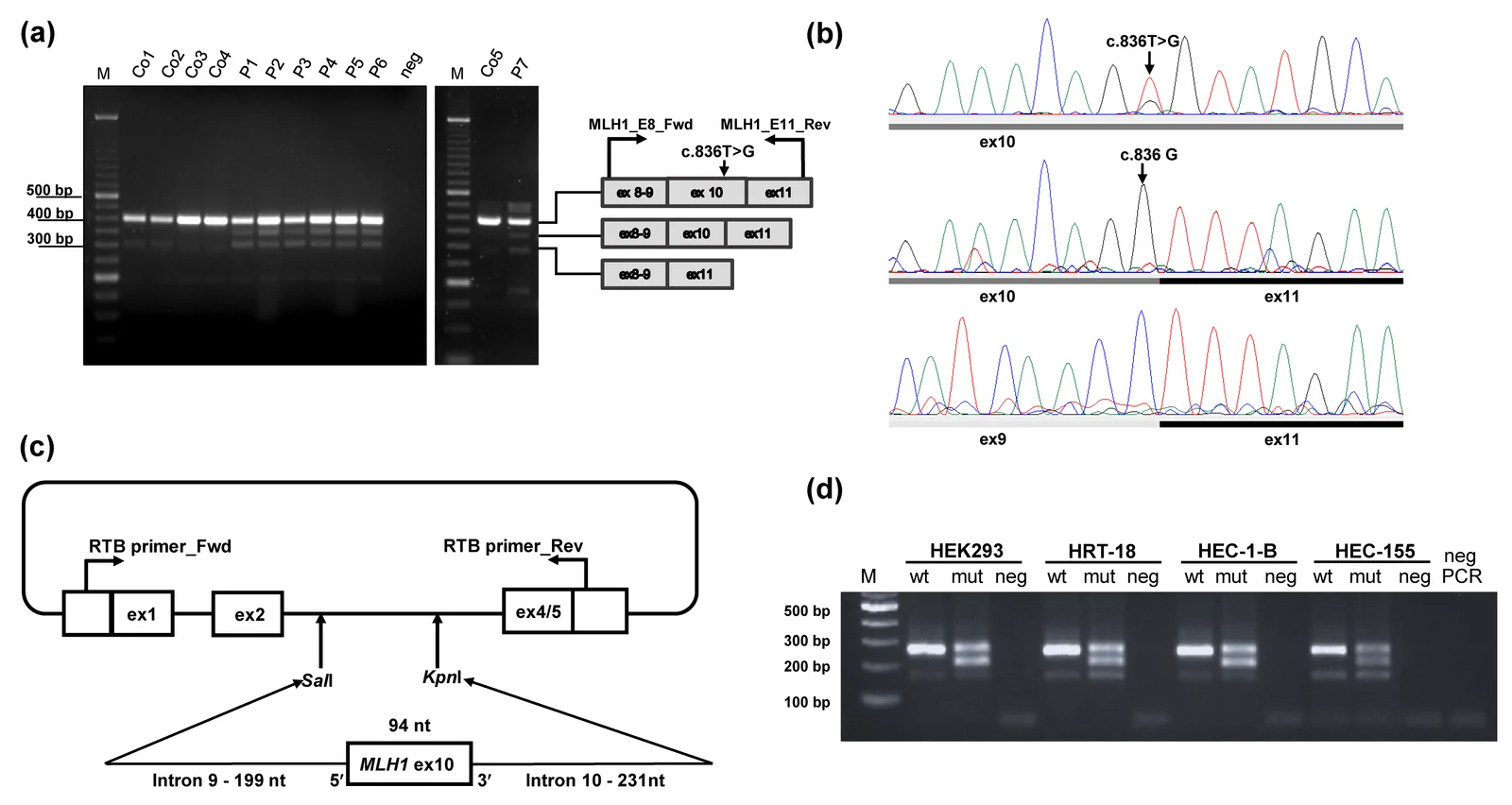
Analysis of patients’ and minigene transcripts show that *MLH1* variant c.836T>G leads to two different splice effects as well as correctly spliced full-length transcripts. (**a**) RT-PCR products of *MLH1* transcripts (the RT-PCR primers MLH1 E8_Fwd and MLH1 E11_Rev are indicated with arrows) from four controls (Co1–4) and the seven patients (P1–P7), showing amplicons of 391, 343, and 297 bp length. A schematic representation of the transcripts from which these amplicons derive is shown on the right side. (**b**) Sanger sequencing of the three amplicons extracted from an agarose gel of the RT-PCR products from patient P1 confirms that these amplicons derive from full-length transcripts (391 bp; (top)), transcripts lacking the last 48 nucleotides of exon 10 (343 bp; (middle)), and transcripts with exon 10 skipping (297 bp; (bottom)). Note that a proportion (~25%) of the full-length transcripts result from the *MLH1* allele with variant c.836T>G. (**c**) Schematic representation of the RTB hybrid-minigene containing *SalI* and *KpnI* restriction sites is shown. Exons (ex) are represented with boxes. The positions of the primers RTB primer_Fwd and RTB primer_Rev used for RT-PCR amplification of the minigene transcripts are indicated with arrows. The 524 bp insert of *MLH1* exon 10 with flanking intronic sequences and *SalI* and *KpnI* restriction sites is shown at the bottom. (**d**) RT-PCR products of transcripts from minigene constructs containing inserts with wildtype (wt) and variant (mut) *MLH1* exon 10 sequences and transiently transfected into the four different cell lines, HEK293, HRT-18, HEC-1-B, and HEC-155, are shown. The size of the bands in the marker (M) is indicated on the left of the gels. No template (water) controls are labeled negative control (neg).

To evaluate by which mechanism *MLH1*:c.836T>G impairs functionality of the gene product, we first tested for a possible splice effect of the variant because especially in silico splice site prediction programs, SSF-like, MaxEntScan, and NNSPLICE, indicated that *MLH1*:c.836T>G creates a strong de novo 5′-splice site 48 bp upstream of the natural one in exon 10 (Supplementary Figure S5). RT-PCR using RNA extracted from puromycin-treated short-term lymphocyte cultures of all seven patients and three healthy controls showed in all patients two aberrant PCR products of 343 bp and 297 bp, respectively, in addition to the 391 bp amplicon, corresponding to the normal full-length transcript amplified with primers in exons 8 and 11. In the controls, the 343 bp PCR product was absent, whereas a faint background band of 297 bp product was also observed (Figure 1a). Sequencing of all three PCR products from one patient showed that the nucleotide exchange r.836u>g, p.(Val279Gly), was present in a proportion (<50%) of the full-length transcripts (Figure 1b). Furthermore, it confirmed that the 343 bp PCR product resulted from transcripts using the novel 5′ splice site created by *MLH1*:c.836T>G instead of the natural one. This leads to skipping of the last 48 nt of exon 10 (r.837_884del), which is predicted to cause replacement of 17 amino acids by a glycine, p.(Val279_Ser295delinsGly). Lastly, it showed that the 297 bp product, which is present in higher abundance in all patients than in the controls, results from transcripts skipping exon 10 (r.791_884del), which is predicted to lead to the premature stop codon p.(His264Leufs*2) and, hence, nonsense-mediated decay (Figure 1b).

To further confirm these splice effects observed in the patients’ transcripts, we performed hybrid-minigene experiments with constructs containing the variant and wildtype *MLH1* exon 10 flanked by intronic sequences (Figure 1c) in four cell lines, HEK293, HRT-18, HEC-1-B, and HEC-155, representing (cancers in) different tissues/organs. The results were in agreement with the observations in the patients’ transcripts. Visual comparison of the PCR products generated from the transcripts of the minigene with the variant c.836T>G to those of the wildtype construct shows consistently across all cell lines that variant c.836T>G leads to (i) a decrease of full-length transcripts (largest PCR product in Figure 1d), (ii) transcripts that lack the last 48 nucleotides of exon 10 (r.837_884del) and that are absent in the wildtype controls, and (iii) a notable increase in the proportion of transcripts skipping exon 10 (r.791_884del; smallest PCR product in Figure 1d).

These results confirm that the *MLH1* variant c.836T>G causes two different deleterious splice effects: (i) use of a newly created 5’splice site in exon 10 leading to loss of, in part, highly conserved 17 amino acids, p.(Val279_Ser295delinsGly), which are located in a MLH1 domain presumed to be part of the MutSα interaction domain [38,39], and (ii) an increase of out-of-frame exon 10 skipping. Of note, these splice effects are *leaky* and a significant proportion of the transcripts from the variant allele are full length, containing the substitution r.836u>g.

### 3.5. MLH1 p.Val279Gly Has a Residual Mismatch Repair Activity

*MLH1* transcripts with substitution r.836u>g are predicted to cause amino acid exchange p.(Val279Gly). This alteration is consistently predicted to be deleterious by the algorithms of MutationTaster, SIFT, PolyPhen2 (PP2), AlphaMissense, and REVEL. In agreement, the combined MAPP/PP2 score of 0.90 (as provided by Priors V2.0) indicates a high probability of pathogenicity for this missense variant [40]. The results of the cell-free in vitro MMR activity (CIMRA) assay performed in a reference laboratory [36], however, indicated that the amino acid change leads to a small reduction of the relative mismatch repair capacity to 80% of that of the wildtype (Supplementary Figure S6), which translates in odds for pathogenicity (OddsPath) of 0.0135 [36].

### 3.6. Family Histories of the Seven Variant Carriers Indicate a Reduced Penetrance of MLH1 Variant c.836T>G

To get information on the penetrance of *MLH1*:c.836T>G, we analyzed clinical and family histories of the seven index patients (Supplementary Figure S7). Excluding patient P4, who also carries a *MSH6* PV and had MSH6-deficient cancers, the age at the first cancer in the six remaining patients ranged from 35 to 74 years with a median age of 52.5 years, which is slightly higher than the previously published 44 years for *MLH1* PV carriers [41]. But it should be noted that the number of patients with *MLH1*:c.836T>G is very small (95% CI ranges actually from 35 to 74 years), and there are also other numbers for the median age of cancer onset in *MLH1* PV carriers, e.g., 48–52 years [42], which align with the median age of cancer onset in our patients.

Analyzing the family history of the seven patients, it is remarkable that only two, patients P2 and P4, have first-degree relatives with a cancer falling in the LS spectrum. Patient P2 has a sibling with CRC aged 47 years. However, this brother was the only one of the patient’s five siblings who did not carry *MLH1*:c.836T>G. Furthermore, his tumor showed loss of MSH2 and MSH6 expression, which resulted likely from purely somatic biallelic inactivation of the *MSH2* gene, as no germline variant in *MSH2* or *MSH6* was found despite extensive analyses, including *MSH2* transcript analysis. The counselling and predictive testing in this family were further complicated by the identification of the *MSH6* VUS (c.2561_2563del), p.(Lys854del), in patient P2, which was also absent in this brother. Both parents of patient P4 had cancer (potentially) falling in the LS spectrum. The mother had CRC at age 64 years and the father had bladder cancer of unknown histology aged 82 years. Since patient P4 carries *MLH1*:c.836T>G and the *MSH6* PV c.3103C>T, p.(Arg1005*), it is possible that one of the parents, who were not available for testing, carried the *MSH6* PV and the other *MLH1*:c.836T>G. The latter would be the only first-degree relative of one of our index patients with *MLH1*:c.836T>G who also might have a LS-associated tumor. The only patient who reported a third-degree relative with LS-related cancer was patient P3, who had a paternal cousin with CRC at age 50 years. He also had a maternal cousin with bladder cancer, but *MLH1*:c.836T>G was paternally inherited, as the patient’s mother tested negative for this variant. Taken together, none of the Tyrolean index patients had a family history that would clearly indicate *MLH1*-associated LS tumors in other family members and none of the nine relatives aged 22–80 years (median age 45 years) who had a positive *MLH1*:c.836T>G testing result had cancer. These data suggest that *MLH1*:c.836T>G could be associated with a lower risk for LS-associated cancers than other *MLH1* (L)PV.

### 3.7. Absence of MLH1 Variant c.836T>G in Unselected Endometrial and Colorectal Cancers of “Tyrolean” Patients

To determine whether the variant is frequent in “Tyroleans” with and without LS-associated tumors, we genotyped tumor tissues of anonymized EC (*n* = 206) and CRC (*n* = 316) of patients with likely “Tyrolean” origin that were available for testing for *MLH1*:c.836T>G. The cancers were not selected for MMRd. The EC DNA was obtained at primary surgery of patients aged 37–93 years (median age 69 years) at diagnosis, and no data on MMR deficiency were assessed [12,13]. The CRC patients had a median age at diagnosis of 69 years (29–94 years). Here, 223 (71%) of analyzed CRCs were MSS, while 45 cases (14%) were MSI with *MLH1* promoter hypermethylation. Forty-eight CRC cases (15%) showed MSI without *MLH1* promotor hypermethylation or unknown methylation data. Five of the 48 tumors (10%) are BRAF V600E mutated, which speaks against LS. Of the remaining 43 patients, 36 exhibited loss of MMR protein(s) expression and four showed expression of all four MMR proteins. IHC data were unavailable for three patients. Thus, 39 patients met the criteria for LS testing [43,44,45,46]. Of these, 20 showed MLH1 and/or PMS2 and 13 MSH2 and/or MSH6 expression loss. The remaining tumors had either atypical IHC pattern (*n* = 3) or IHC data were unavailable (Supplementary Table S2). TaqMan genotyping did not detect *MLH1*:c.836T>G in any of the EC and CRC samples.

## 4. Discussion

We identified the Tyrolean founder variant *MLH1*:c.836T>G in 6/59 (10.2%) of suspected Tyrolean LS patients with MLH1 and/or PMS2 expression loss in their carcinoma, and in overall 3.5% (7/200) suspected Tyrolean LS patients analyzed within a timeframe of 15.5 years. Among a total of 42 different MMR gene variants causing LS in 63 patients of our cohort, it is the second most frequent (Supplementary Table S1). These numbers illustrate that this Tyrolean founder variant is an important cause of LS-associated cancer in the Tyrolean population. This notwithstanding, the formal classification of this variant posed and still poses major obstacles.

In ClinVar, *MLH1*:c.836T>G is listed only five times with classifications ranging from likely benign (LB) to pathogenic (P). The evidence criteria given for classification of the variant as LB were summarized by Tsai et al. [37]. They are a prior probability of 0.1 calculated at that time from a PolyPhen-2 score of 0.99 and a MAPP score of 2.32 [47], co-segregation analysis in one family, and additional arguments that resulted in a posterior probability of pathogenicity of 0.018, which is consistent with LB [48]. The main argument put forward by the commercial laboratory Labcorp Genetics (formerly Invitae) who deposited the variant to ClinVar as P is that their internal RNA analysis showed that the variant leads to *MLH1* exon 10 and/or exon 10–11 skipping, both having a truncating effect, p.(His264Leufs*2) and p.(Arg265Phefs*14), respectively. These arguments for and against the pathogenicity of the variant need to be put into perspective of findings presented in this study. Applying the widely accepted ACMG/AMP Variant Interpretation Guidelines specified for *MLH1* variants in the current version 2.0.0 of the ClinGen InSiGHT Hereditary Colorectal Cancer/Polyposis Expert Panel Specifications (in the following referred to as the VCEP MLH1 guidelines), conflicting classification evidence needs to be taken into account. We also show that *MLH1*:c.836T>G has a splice effect. However, we observed only a slight increase of the out-of-frame exon 10 skipping in carriers, especially when compared to non-carrier controls, in whom exon 10 skipping was also detectable. Leaky exon 10 skipping has been reported as an effect also of other single nucleotide variants in exon 10 that do not affect nucleotides of the consensus splice sites [49]. The main splice effect of *MLH1*:c.836T>G not observed in controls is the use of a newly created 5′ splice site in exon 10 leading to in-frame loss of 48 nucleotides predicted to cause the replacement of 17 amino acids with a glycine, p.(Val279_Ser295delinsGly), in a domain presumed to be part of the MLH1 MutSα interaction domain. Although this main splice effect would alter a region that is defined as “critical for protein function”, the ACMG/AMG evidence criterion PVS1 cannot be applied because this splice effect is “leaky”, as there are clearly full-length transcripts containing a G at position c.836 in patient-derived mRNA, and in ex vivo experiments full-length transcripts are transcribed from the variant minigene construct (Figure 1). The missense effect of p.Val279Gly is predicted by nearly all in silico programs (REVEL, Align GVGD, AlphaMissense, MutationTaster, PolyPhen-2, and SIFT) to affect functionality and, most importantly, has a MAPP/PP2 combined score of 0.90 and, thus, a HCI prior probability for pathogenicity > 0.81, which allows us to use the ACMG/AMG evidence criterion PP3 at strength moderate (PP3_moderate). However, the CIMRA assay, one of the calibrated assays recommended by the VCEP MLH1 guidelines, performed in a reference laboratory showed that this amino acid change decreases the MLH1 mismatch repair capacity only by 20% (Supplementary Figure S3), translating in odds for pathogenicity (OddsPath) of 0.0135 [36]. This (i.e., functional OddsPath ≤ 0.05) is a strong evidence criterion against pathogenicity (BS3_strong) of this missense effect contradicting evidence criterion PP3_moderate. The strongest evidence criterion in favor of pathogenicity for this variant derives from the associated phenotype. Since more than three, i.e., four, index patients with this variant had CRC showing MLH1 deficiency in the absence of *MLH1* promoter hypermethylation (Table 2), the evidence criterion PP4 at category strong (PP4_strong) may be used for classification. The only patient whose tumors did not show MLH1 and/or PMS2, but MSH6 deficiency, had in addition to the *MLH1* founder variant also the *MSH6* PV c.3013C>T, p.(Arg1005*). However, a single patient with an alternate molecular basis for LS is insufficient for applying the corresponding evidence criterion BP5 against pathogenicity. Finally, absence of the variant in gnomAD is another supporting evidence criterion (PM2_supporting) for its pathogenicity. Taken together, our data render, according to the VCEP MLH1 guidelines, one strong (PP4_strong), one moderate (PP3_moderate), and one supporting (PM2_supporting) pathogenic evidence, as well as one strong benign evidence (BP3_strong). To combine contradictory evidence criteria, Tavtigian et al. developed the Bayesian Equation (5) to calculate OddsPath [48]. Using the simple spreadsheet calculator provided in the Supplementary Materials to this paper, the evidence criteria render a combined OddsPath of 9.00, which equals a posterior probability of 0.5 for pathogenicity and, hence, a classification as VUS. The same classification is reached using the point system that essentially recapitulates the Bayesian formulation of the ACMG/AMG guidelines [50] and renders three points (1 + 2 + 4 – 4 = 3).

Despite that formal classification of *MLH1*:c.836T>G as (L)P is not possible using the current internationally accepted VCEP MLH1 guidelines, our data of six carriers clearly show that this variant is associated with MLH1-deficient tumors occurring in patients as young as 35 years. Furthermore, we show loss of the wildtype *MLH1* allele in at least one tumor, indicating that *MLH1*:c.836T>G is directly involved in MMR deficiency of this tumor. Hence, our data strongly indicate that this variant increases the risk for young-onset LS-associated tumors. We informed patients and their referring clinicians about our findings by reporting this variant as LP. We also offered predictive testing to their relatives. However, it is noteworthy that the two factors that seem to prevent the formal classification of *MLH1*:c.836T>G as (L)P with the current VCEP MLH1 guidelines, i.e., (i) the leakiness of its splice effect and (ii) the small effect of the amino acid change on the functionality of the protein in a functional assay, are often associated with variants of lower penetrance (see further discussion below). Hence, it is possible that the obstacles to formal classification of this variant as (L)P are related to a reduced penetrance and, consequently, a reduced cancer risk of carriers. In support of this, we note that none of the six index patients with LS due to this variant had a strong family history. Furthermore, both carcinomas in patient P4, who is heterozygous also for the *MSH6* PV p.(Arg1005*), were MSH6-deficient, although in general *MLH1* PV are more penetrant than *MSH6* PV. Taken together, these findings suggest that *MLH1*:c.836T>G could have lower penetrance compared to an average *MLH1* PV. However, the number of families identified in our cohort is too small to perform meaningful penetrance calculations. Also, genotyping two anonymized cohorts of EC (*n* = 206) and CRC (*n* = 316), including 20 CRC with MLH1/PMS2 expression loss without *MLH1* promoter hypermethylation, from patients with likely Tyrolean origin was unable to provide data to corroborate this assumption. As the actual penetrance and frequency of this Tyrolean founder mutation are currently unknown, we suggest that carriers undergo cancer surveillance as is recommended for carriers of *MLH1* PV in general until further data on the penetrance of this variant become available. We advise patients to regularly request updated information regarding the penetrance of this variant to adjust their surveillance strategies if indicated.

*MLH1*:c.836T>G appears to represent an old Tyrolean founder variant (estimated age 2725–7950 years). Of note, in Tyrol, an elevated frequency (8.6%) of the Y-chromosomal haplogroup G-L497 has been observed. This haplogroup is a European subclade within G2a/G-M201, a lineage repeatedly associated with early Neolithic farmers in Europe [51]. It shows a clustered distribution in Tyrol with high frequencies (up to 40%) specifically in some side valleys of the Tyrolean upper Inn Valley [51], where 4/7 carriers of the variant live. These observations are compatible with long-term regional ancestry and local founder effects in these valleys. Hence, reliable regional frequency assessments of *MLH1*:c.836T>G in the general population and in patients with LS-associated tumors may allow us to achieve better penetrance estimates.

Taking a broader view on the classification of variants, our analysis of the Tyrolean founder variant shows that while the ACMG/AMP guidelines for variant interpretation and their VCEP specifications were important steps toward standardizing, harmonizing, and, hence, improving variant classification, their application insufficiently classifies variants with (potentially) reduced penetrance. While the example of *MLH1*:c.836T>G illustrates that with the current evidence criteria (possible) low-penetrance alleles cannot be classified as (L)P, there are, on the other side, examples of such variants that have been classified as (L)P. One example is NM_000179.3 (*MSH6*):c.3226C>T identified in our cohort in one patient with CRC at age 42 without family history of LS-associated cancer. This missense variant p.(Arg1076Cys) is classified as LP by the VCEP expert panel mainly based on clinical evidence criteria, as it was found in several patients with MSH6-deficient cancers (PP4_strong) and caused autosomal recessive constitutional MMR deficiency (CMMRD) syndrome in four patients in whom it was found in compound heterozygosity with different truncating *MSH6* variants and who had MMR deficiency in normal cells (current criterion PM3_strong) [52,53,54]. Identification of a MMR gene variant in homozygosity or compound heterozygosity in individuals with CMMRD, a phenotype that is clinically characterized by cancer onset in the first two decades of life (median age of onset < 10 years) [55] and molecularly by MMR deficiency in non-neoplastic cells, i.e., constitutional MSI [56], confirms that the variant impairs function and increases cancer risk. However, attenuated forms of this condition may also unveil that a variant has reduced penetrance. Therefore, it is noteworthy that individuals who carry missense variant p.(Arg1076Cys) homozygously manifest with a LS-like rather than a CMMRD clinical phenotype, which, in combination with lower scores of constitutional MMR deficiency, strongly suggests that this variant has reduced penetrance [57]. To the best of our knowledge, *MSH6* variant p.(Arg1076Cys) is not yet tested in the CIMRA assay, but in a recently published novel functional assay it showed impaired functionality with a calculated OddPath score that would translate into a strong evidence category (PS3) [58]. Yet, in agreement with the reduced penetrance of this *MSH6* variant, its OddPath score is by far the lowest of all positive variants tested with this novel assay.

Examples of low-penetrance/hypomorphic variants that cause difficulties in appropriate classification because they impair functionality through a deleterious, albeit leaky, splice effect are the well-known founder variant *PMS2*:c.2002A>G (r.[2002_2006del,2002A>G]; p.[(Ile668*,Ile668Val)]), which is responsible for several cases of attenuated CMMRD among Innuits from Nunavik (Quebec, Canada) [59], and *MLH1*:c.306G>A (r.[208_306del, 306G>A]; p.[(Lys70_Glu102del,Glu102=)]), also found with attenuated CMMRD [57]. Both variants are classified and deposited by most laboratories as (L)P to ClinVar, while they are so far unclassified or classified as VUS by the VCEP consortium. In addition to *MLH1*:c.836T>G, we found in our cohort of suspected LS patients the leaky splice variant *PMS2*:c.255G>A (r.[251_353del,255G>A]; p.[Leu85Metfs*17;Leu85=]) in a 48-year-old patient with PMS2-deficient EC. This variant is classified by most laboratories as (L)B because of the high allele frequency (Grpmax Filtering AF: >0.0001; BS1_strong) and no predicted splice effect of this silent variant. However, as holds true for *MLH1*:c.836T>G, also for this *PMS2* variant SpliceAI delta scores did neither speak in favor of nor against a splice effect, so that neither the evidence criteria PP3 nor BP4/BP7 can be applied and the variant has to be classified as VUS, especially because our case has a PMS2-deficient EC, which allows us to also use evidence criterion PP4_supporting. In agreement, we reported it as VUS and did not offer predictive testing to relatives. Nonetheless, it would not be surprising if this variant, like *MLH1*:c.306G>A, would cause (attenuated) CMMRD in individuals compound heterozygous or homozygous for this variant. *MSH6*:c.2561_2563del, p.(Lys854del), which we identified in patient P2 in addition to *MLH1*:c.836T>G, is yet another potentially hypomorphic variant that causes CMMRD in compound heterozygosity with another *MSH6* (L)PV [60,61]. However, according to the applicable evidence categories (PM2_supporting and PM3_moderate), it has to be classified as VUS according to the VCEP MSH6 guidelines.

Taken together, *MLH1*:c.836T>G and other (possible) low-penetrance/hypomorphic MMR variants illustrate that (possible) low-penetrance MMR gene variants are insufficiently categorized in a binary system of pathogenic and benign variants and potentially need to be differently classified in the context of the autosomal recessive CMMRD syndrome than for LS. In addition, a different framework for the evaluation of low-penetrance/hypomorphic risk alleles should be considered. Whether adjustments to the current VCEP MLH1 guidelines, such as allowing for the use of the ACMG/AMG evidence criterion PVS1_RNA also for variants with a leaky splice effect, albeit at lower strength, as has been proposed, e.g., by the CanVIG-UK group [62], or whether other classification systems, e.g., the Sherloc system [63] or the ABC system [64,65], that have been proposed to be used in addition or alternatively to the ACMG/AMP system, can be helpful in this respect needs to be evaluated in a larger effort, as it is beyond the scope of this report.

## 5. Conclusions

Our extensive study of the leaky splice variant *MLH1*:c.836T>G shows that although its formal classification as (L)P is not possible using the current internationally accepted VCEP MLH1 guidelines, it clearly increases the risk for LS-associated tumors. Hence, this variant should be reported to patients. It should be explained that the penetrance of this variant is currently uncertain. Therefore, until further data of its penetrance become available, we recommend cancer surveillance, as suggested for all carriers of *MLH1* (L)PV. Furthermore, the examples of this and other (possible) low-penetrance/hypomorphic variants illustrate the need for a better terminology for (potential) low-penetrance MMR gene variants, as their classification as VUSs is unsatisfactory. Therefore, we hope that this report is also seen as a plea to install international expert task forces to develop guidelines for appropriate classification and evaluation of low-penetrance/hypomorphic MMR gene variants.

## Figures and Tables

**Table 1:** Clinicopathological features of 200 Tyrolean-suspected LS patients.

Variable	Number of Patients/ Tumors (%)
Gender *	
Male	64 (32%)
Female	136 (68%)
**Cancers in 190 patients**	
Intestinal tract Ca ^$^	96 (50.5%)
EC, OC ^$^	52 (27.4%)
Other LS-associated Ca ^$,¥^	20 (10.5%)
>1 LS-associated Ca ^$^	12 (6.3%)
≥1 non-LS Ca	10 (5.3%)
**Age at first cancer diagnosis**	
<50 yr	57 (28.5%)
50–60 yr	66 (33%)
>60 yr	64 (32%)
N/D	3 (1.5%)
No cancer/family history LS and/or polyps	10 (5%)
**Microsatellite status ^#^**	
MSI	81 (42%)
MSS	17 (9%)
N/D	94 (49%)
**Loss of MMR protein expression ^#^**	
MLH1/PMS2: −/−	41 (21.3%)
MLH1/PMS2: +/−	18 (9.4%)
MSH2/MSH6: −/−	18 (9.4%)
MSH2/MSH6: +/−	17 (8.9%)
Atypical pattern	9 (4.7%)
MLH1/MSH2/MSH6/PMS2: +/+/+/+	24 (12.2%)
N/D or incomplete data	65 (33.8%)
***MLH1*** **promotor methylation in MLH1-deficient samples ^§^**	
Unmethylated	17 (41.5%)
Hypermethylated	3 (7.3%)
N/D	21 (51.2%)

*, Data not included in Supplementary Table S1; ^$^, with and without non-LS-associated cancers; ^¥^, bile duct, gastric, urothelial, and sebaceous cancer; ^#^, microsatellite status and MMR protein expression results of 192 Tyrolean-suspected LS patients with tumors are taken into account; ^§^, tumors with missing or atypical data were omitted. Abbreviations: LS, Lynch syndrome; Ca, cancer; EC, endometrial cancer; OC, ovarian cancer; yr, year; MSI, microsatellite instable; MSS, microsatellite stable; −, loss of protein expression; +, normal protein expression.

**Table 2:** Clinicopathological characteristics of seven Tyrolean-suspected LS patients carrying *MLH1* variant c.836T>G.

Index	Gender	Dx	Age at Dx (yr)	MS Status	Expression of MLH1/PMS2/MSH2/MSH6	*MLH1* Promotor Hypermethylation	BRAF V600E	LOH/ Somatic *MLH1* PV
P1	F	CRC	39	MSI	−/−/+/+	neg	wt	LOH
P2	F	CRC	35	MSI	−/−/+/+	neg	wt	N/E
P3	M	CRC	55	MSI	+/−/+/+	neg	wt	N/E
P4 *	F	EC, UC	59, 64	MSS ^§^	+/+/+/−	n.a.	n.a.	n.a.
P5	M	CRC	50	MSI	−/−/+/+	neg	wt	N/E
P6	M	SBC	59	MSI	−/−/+/+	neg	wt	N/E
P7	M	CRC	74	MSI	−/−/+/+	neg	wt	somatic PV ^‡^

*, PV MSH6:c.3103C>T, p.(Arg1005*), was also identified; ^§^, results only of endometrial tumor; ^‡^, MLH1:c.1191_1192delinsTT, p.(Gln398*) found with a VAF of 24% in tumors. Abbreviations: F, female; M, male; CRC, colorectal cancer; EC, endometrial cancer; UC, urothelial cancer; SBC, small bowel (intestine) cancer; Dx, diagnosis; yr, year; MS, microsatellite; MSI, microsatellite instable; MSS, microsatellite stable; −, expression loss; +, normal protein expression; neg, negative; n.a., not applicable; wt, wildtype; LOH, loss of heterozygosity; PV, pathogenic variant; N/E, data not evaluable.

**Table 3:** Common haplotype around *MLH1* variant c.836T>G in seven Tyrolean LS patients.

SNP ID	Position chr3: (GRCh37/hg19)	P1	P2	P3	P4	P5	P6	P7	Shared Allele
rs73054203	36729011	A A	A B	A B	A B	A B	B B	A B	-
rs954494	36749352	A A	A A	A A	A B	A B	B B	A B	-
rs2063159	36777041	A B	A B	A B	A B	A B	B B	A A	-
rs7632683	36794597	A B	AB	A B	A B	A B	B B	A A	-
rs9820240	36796647	B B	A B	B B	B B	A B	A B	B B	B
rs67994182	36832949	A A	A B	A B	A B	A B	A B	A B	A
rs4441609	36892717	A B	B B	A B	B B	B B	B B	A B	B
rs6777217	36979042	A A	A B	A A	A B	A B	A A	A A	A
rs1799977 *	37053568	A A	A B	A A	A B	A A	A A	A A	A
rs10849	37095070	B B	A B	B B	A B	B B	B B	B B	B
rs147872149	37217044	B B	A B	B B	A B	B B	B B	B B	B
rs4452278	37346319	B B	A B	B B	A B	B B	B B	B B	B
rs1029236	37474256	A A	A B	A A	A A	A A	A A	A A	A
rs267561	37574951	B B	B B	B B	B B	B B	A B	AB	B
rs79305345	37669707	A B	B B	B B	B B	B B	B B	B B	B
rs77147659	37783896	A B	A B	A B	A B	A B	B B	A B	B
rs2507948	37832172	A A	A B	A B	A B	A B	A A	A A	A
rs115894276	37866662	B B	B B	B B	B B	B B	B B	B B	B ^#^
rs13077801	37870175	A B	B B	A A	A A	A A	A B	A A	-
rs73058943	37980724	A A	B B	A B	A B	A A	B B	A B	-
rs1384006	38261037	A B	A B	A A	A A	A A	B B	A B	-

From the SNPs with gray underlay a common haplotype of all seven patients can be deduced. The size of the maximum common haplotype of approximately 1.075 Mb is delineated by SNPs rs7632683 and rs13077801; *, the SNP rs1799977 is in *MLH1* (chr3: 37035009–37092337); ^#^, not informative SNP. Abbreviations: chr, chromosome; SNP, single nucleotide polymorphism.

## Data Availability

The original contributions presented in this study are included in the article/Supplementary Materials. The variant NM_000249.3(MLH1):c.836T>G is deposited in ClinVar. Further inquiries can be directed to the corresponding author.

## Supplementary Materials

The following supporting information can be downloaded at: https://www.mdpi.com/article/10.3390/biom16081200/s1, Figure S1: Immunohistochemical (IHC) staining of the four mismatch repair (MMR) proteins in colorectal cancer tissue from patient P3 shows PMS2 expression loss with intact expression of the other MMR proteins. Figure S2: Immunohistochemical (IHC) staining of the four MMR proteins in small bowel (intestine) cancer of patient P6 shows MLH1 and PMS2 expression loss with intact expression of the proteins MSH2 and MSH6. Figure S3: Immunohistochemical (IHC) staining of the four MMR proteins in endometrial cancer tissue of patient P4 shows MSH6 expression loss with intact expression of the other MMR proteins. Figure S4: Sanger sequencing of the *MLH1* variant c.836T>G and flanking nucleotides in DNA from blood (upper panel) and tumor DNA (lower panel) from patient P1. Figure S5: Graphical representation of the predicted splice effect of *MLH1* variant c.836T>G. Figure S6: Relative repair efficiency of MLH1 missense variant p.Val279Gly. Figure S7: Pedigree of seven probands. Table S1: Detailed clinicopathological features of 200 unrelated Tyrolean-suspected LS patients. Table S2: Tumor features observed in the unselected Tyrolean colorectal cancer (CRC) cohort (*n* = 316). Table S3: List of PCR primers and sequences. Table S4: Original input data (maximal common haplotype patients P1–P7) with addition information in Genetic Mutation Age Estimator. Table S5: Mutation age in generations with CI. Table S6: Mutation age in years (assuming one generation = 25 years) with CI.

## Abbreviations

bp	base pair
°C	degrees Celsius
cDNA	complementary DNA
CI	confidence interval
CIMRA	cell-free in vitro MMR activity
cM	centimorgan
CMMRD	constitutional mismatch repair deficiency
CN	copy number
CRC	colorectal cancer
DNA	deoxyribonucleic acid
EC	endometrial cancer
FFPE	formalin-fixed paraffin-embedded
gDNA	genomic DNA
IHC	immunohistochemical
(L)PV	(likely) pathogenic variant
LB	likely benign
LOH	loss of heterozygosity
LS	Lynch syndrome
Mb	megabase
MGB	minor groove binding
MLPA	multiplex ligation-dependent probe amplification
MMR	mismatch repair
MMRd	mismatch repair deficiency
mRNA	messenger RNA
MSI	microsatellite instability
MSS	microsatellite stability
MUI	Medical University Innsbruck
NGS	next-generation sequencing
nt	nucleotide
OC	ovarian cancer
OddsPath	odds for pathogenicity
P	pathogenic
PCR	polymerase chain reaction
PHA	phytohemagglutinin
PMR	percentage of methylated reference
PV	pathogenic variant
RNA	ribonucleic acid
RT-PCR	reverse transcription polymerase chain reaction
SNP	single nucleotide polymorphism
VAF	variant allele frequency
VCEP	variant curation expert panel
VUS	variant of uncertain significance

## References

[1] Win A.K., Jenkins M.A., Dowty J.G., Antoniou A.C., Lee A., Giles G.G., Buchanan D.D., Clendenning M., Rosty C., Ahnen D.J. (2017). Prevalence and Penetrance of Major Genes and Polygenes for Colorectal Cancer. Cancer Epidemiol. Biomark. Prev..

[2] Park J., Karnati H., Rustgi S.D., Hur C., Kong X.F., Kastrinos F. (2025). Impact of population screening for Lynch syndrome insights from the All of Us data. Nat. Commun..

[3] Yurgelun M.B., Kulke M.H., Fuchs C.S., Allen B.A., Uno H., Hornick J.L., Ukaegbu C.I., Brais L.K., McNamara P.G., Mayer R.J. (2017). Cancer Susceptibility Gene Mutations in Individuals with Colorectal Cancer. J. Clin. Oncol..

[4] Ryan N.A.J., Glaire M.A., Blake D., Cabrera-Dandy M., Evans D.G., Crosbie E.J. (2019). The proportion of endometrial cancers associated with Lynch syndrome: A systematic review of the literature and meta-analysis. Genet. Med..

[5] Seppala T.T., Latchford A., Negoi I., Sampaio Soares A., Jimenez-Rodriguez R., Sanchez-Guillen L., Evans D.G., Ryan N., Crosbie E.J., Dominguez-Valentin M. (2021). European guidelines from the EHTG and ESCP for Lynch syndrome: An updated third edition of the Mallorca guidelines based on gene and gender. Br. J. Surg..

[6] Ten Broeke S.W., van der Klift H.M., Tops C.M.J., Aretz S., Bernstein I., Buchanan D.D., de la Chapelle A., Capella G., Clendenning M., Engel C. (2018). Cancer Risks for PMS2-Associated Lynch Syndrome. J. Clin. Oncol..

[7] Dominguez-Valentin M., Sampson J.R., Seppala T.T., Ten Broeke S.W., Plazzer J.P., Nakken S., Engel C., Aretz S., Jenkins M.A., Sunde L. (2020). Cancer risks by gene, age, and gender in 6350 carriers of pathogenic mismatch repair variants: Findings from the Prospective Lynch Syndrome Database. Genet. Med..

[8] Peltomaki P. (2003). Role of DNA mismatch repair defects in the pathogenesis of human cancer. J. Clin. Oncol..

[9] Lynch H.T., de la Chapelle A. (2003). Hereditary colorectal cancer. N. Engl. J. Med..

[10] Gallon R., Gawthorpe P., Phelps R.L., Hayes C., Borthwick G.M., Santibanez-Koref M., Jackson M.S., Burn J. (2021). How Should We Test for Lynch Syndrome? A Review of Current Guidelines and Future Strategies. Cancers.

[11] Walker L.C., Hoya M., Wiggins G.A.R., Lindy A., Vincent L.M., Parsons M.T., Canson D.M., Bis-Brewer D., Cass A., Tchourbanov A. (2023). Using the ACMG/AMP framework to capture evidence related to predicted and observed impact on splicing: Recommendations from the ClinGen SVI Splicing Subgroup. Am. J. Hum. Genet..

[12] Degasper C., Brunner A., Sampson N., Tsibulak I., Wieser V., Welponer H., Marth C., Fiegl H., Zeimet A.G. (2019). NADPH oxidase 4 expression in the normal endometrium and in endometrial cancer. Tumour Biol..

[13] Wieser V., Abdel Azim S., Sprung S., Knoll K., Kogl J., Hackl H., Marth C., Zeimet A.G., Fiegl H. (2020). TNFα signalling predicts poor prognosis of patients with endometrial cancer. Carcinogenesis.

[14] Etzler J., Peyrl A., Zatkova A., Schildhaus H.U., Ficek A., Merkelbach-Bruse S., Kratz C.P., Attarbaschi A., Hainfellner J.A., Yao S. (2008). RNA-based mutation analysis identifies an unusual MSH6 splicing defect and circumvents PMS2 pseudogene interference. Hum. Mutat..

[15] Wernstedt A., Valtorta E., Armelao F., Togni R., Girlando S., Baudis M., Heinimann K., Messiaen L., Staehli N., Zschocke J. (2012). Improved multiplex ligation-dependent probe amplification analysis identifies a deleterious PMS2 allele generated by recombination with crossover between PMS2 and PMS2CL. Genes Chromosom. Cancer.

[16] Povysil G., Tzika A., Vogt J., Haunschmid V., Messiaen L., Zschocke J., Klambauer G., Hochreiter S., Wimmer K. (2017). panelcn.MOPS: Copy-number detection in targeted NGS panel data for clinical diagnostics. Hum. Mutat..

[17] den Dunnen J.T., Antonarakis S.E. (2000). Mutation nomenclature extensions and suggestions to describe complex mutations: A discussion. Hum. Mutat..

[18] Ioannidis N.M., Rothstein J.H., Pejaver V., Middha S., McDonnell S.K., Baheti S., Musolf A., Li Q., Holzinger E., Karyadi D. (2016). REVEL: An Ensemble Method for Predicting the Pathogenicity of Rare Missense Variants. Am. J. Hum. Genet..

[19] Sim N.L., Kumar P., Hu J., Henikoff S., Schneider G., Ng P.C. (2012). SIFT web server: Predicting effects of amino acid substitutions on proteins. Nucleic Acids Res..

[20] Schwarz J.M., Cooper D.N., Schuelke M., Seelow D. (2014). MutationTaster2: Mutation prediction for the deep-sequencing age. Nat. Methods.

[21] Adzhubei I.A., Schmidt S., Peshkin L., Ramensky V.E., Gerasimova A., Bork P., Kondrashov A.S., Sunyaev S.R. (2010). A method and server for predicting damaging missense mutations. Nat. Methods.

[22] Zhang M.Q. (1998). Statistical features of human exons and their flanking regions. Hum. Mol. Genet..

[23] Yeo G., Burge C.B. (2004). Maximum entropy modeling of short sequence motifs with applications to RNA splicing signals. J. Comput. Biol..

[24] Reese M.G., Eeckman F.H., Kulp D., Haussler D. (1997). Improved splice site detection in Genie. J. Comput. Biol..

[25] Pertea M., Lin X., Salzberg S.L. (2001). GeneSplicer: A new computational method for splice site prediction. Nucleic Acids Res..

[26] Cartegni L., Wang J., Zhu Z., Zhang M.Q., Krainer A.R. (2003). ESEfinder: A web resource to identify exonic splicing enhancers. Nucleic Acids Res..

[27] Perez-Carbonell L., Alenda C., Paya A., Castillejo A., Barbera V.M., Guillen C., Rojas E., Acame N., Gutierrez-Avino F.J., Castells A. (2010). Methylation analysis of MLH1 improves the selection of patients for genetic testing in Lynch syndrome. J. Mol. Diagn..

[28] Gandolfo L.C., Bahlo M., Speed T.P. (2014). Dating rare mutations from small samples with dense marker data. Genetics.

[29] Wimmer K., Schamschula E., Wernstedt A., Traunfellner P., Amberger A., Zschocke J., Kroisel P., Chen Y., Callens T., Messiaen L. (2020). AG-exclusion zone revisited: Lessons to learn from 91 intronic NF1 3′ splice site mutations outside the canonical AG-dinucleotides. Hum. Mutat..

[30] Cooper T.A. (2005). Use of minigene systems to dissect alternative splicing elements. Methods.

[31] Ryan K.J., Cooper T.A. (1996). Muscle-specific splicing enhancers regulate inclusion of the cardiac troponin T alternative exon in embryonic skeletal muscle. Mol. Cell Biol..

[32] Graham F.L., Smiley J., Russell W.C., Nairn R. (1977). Characteristics of a human cell line transformed by DNA from human adenovirus type 5. J. Gen. Virol..

[33] Tompkins W.A.F., Watrach A.M., Schmale J.D., Schultz R.M., Harris J.A. (1974). Cultural and antigenic properties of newly established cell strains derived from adenocarcinomas of the human colon and rectum. JNCI J. Natl. Cancer Inst..

[34] Kuramoto H., Hamano M., Imai M. (2002). HEC-1 cells. Hum. Cell.

[35] Iida T., Hamano M., Yoshida N., Yonamine K., Hayashi K., Kiguchi K., Ishizuka B., Nishimura Y., Arai T., Kawaguchi M. (2004). Establishment and characterization of two cell lines (HEC-155, HEC-180) derived from uterine papillary serous adenocarcinoma. Eur. J. Gynaecol. Oncol..

[36] Drost M., Tiersma Y., Thompson B.A., Frederiksen J.H., Keijzers G., Glubb D., Kathe S., Osinga J., Westers H., Pappas L. (2019). A functional assay-based procedure to classify mismatch repair gene variants in Lynch syndrome. Genet. Med..

[37] Tsai G.J., Ranola J.M.O., Smith C., Garrett L.T., Bergquist T., Casadei S., Bowen D.J., Shirts B.H. (2019). Outcomes of 92 patient-driven family studies for reclassification of variants of uncertain significance. Genet. Med..

[38] Plotz G., Raedle J., Brieger A., Trojan J., Zeuzem S. (2003). N-terminus of hMLH1 confers interaction of hMutLα and hMutLβ with hMutSα. Nucleic Acids Res..

[39] Plotz G., Welsch C., Giron-Monzon L., Friedhoff P., Albrecht M., Piiper A., Biondi R.M., Lengauer T., Zeuzem S., Raedle J. (2006). Mutations in the MutSα interaction interface of MLH1 can abolish DNA mismatch repair. Nucleic Acids Res..

[40] Thompson B.A., Greenblatt M.S., Vallee M.P., Herkert J.C., Tessereau C., Young E.L., Adzhubey I.A., Li B., Bell R., Feng B. (2013). Calibration of multiple in silico tools for predicting pathogenicity of mismatch repair gene missense substitutions. Hum. Mutat..

[41] Talbot A., O’Donovan E., Berkley E., Nolan C., Clarke R., Gallagher D. (2021). The contribution of Lynch syndrome to early onset malignancy in Ireland. BMC Cancer.

[42] Peltomaki P., Nystrom M., Mecklin J.P., Seppala T.T. (2023). Lynch Syndrome Genetics and Clinical Implications. Gastroenterology.

[43] Giardiello F.M., Allen J.I., Axilbund J.E., Boland C.R., Burke C.A., Burt R.W., Church J.M., Dominitz J.A., Johnson D.A., Kaltenbach T. (2014). Guidelines on genetic evaluation and management of Lynch syndrome: A consensus statement by the US Multi-Society Task Force on colorectal cancer. Gastroenterology.

[44] Umar A., Boland C.R., Terdiman J.P., Syngal S., de la Chapelle A., Ruschoff J., Fishel R., Lindor N.M., Burgart L.J., Hamelin R. (2004). Revised Bethesda Guidelines for hereditary nonpolyposis colorectal cancer (Lynch syndrome) and microsatellite instability. J. Natl. Cancer Inst..

[45] Vasen H.F., Mecklin J.P., Khan P.M., Lynch H.T. (1991). The International Collaborative Group on Hereditary Non-Polyposis Colorectal Cancer (ICG-HNPCC). Dis. Colon Rectum.

[46] Vasen H.F., Watson P., Mecklin J.P., Lynch H.T. (1999). New clinical criteria for hereditary nonpolyposis colorectal cancer (HNPCC, Lynch syndrome) proposed by the International Collaborative group on HNPCC. Gastroenterology.

[47] Thompson B.A., Goldgar D.E., Paterson C., Clendenning M., Walters R., Arnold S., Parsons M.T., Michael D.W., Gallinger S., Haile R.W. (2013). A multifactorial likelihood model for MMR gene variant classification incorporating probabilities based on sequence bioinformatics and tumor characteristics: A report from the Colon Cancer Family Registry. Hum. Mutat..

[48] Tavtigian S.V., Greenblatt M.S., Harrison S.M., Nussbaum R.L., Prabhu S.A., Boucher K.M., Biesecker L.G. (2018). on behalf of the ClinGen Sequence Variant Interpretation Working Group. Modeling the ACMG/AMP variant classification guidelines as a Bayesian classification framework. Genet. Med..

[49] Soukarieh O., Gaildrat P., Hamieh M., Drouet A., Baert-Desurmont S., Frebourg T., Tosi M., Martins A. (2016). Exonic Splicing Mutations Are More Prevalent than Currently Estimated and Can Be Predicted by Using in Silico Tools. PLoS Genet..

[50] Tavtigian S.V., Harrison S.M., Boucher K.M., Biesecker L.G. (2020). Fitting a naturally scaled point system to the ACMG/AMP variant classification guidelines. Hum. Mutat..

[51] Berger B., Niederstatter H., Erhart D., Gassner C., Schennach H., Parson W. (2013). High resolution mapping of Y haplogroup G in Tyrol (Austria). Forensic Sci. Int. Genet..

[52] Okkels H., Sunde L., Lindorff-Larsen K., Thorlacius-Ussing O., Gandrup P., Lindebjerg J., Stubbeteglbjaerg P., Oestergaard J.R., Nielsen F.C., Krarup H.B. (2006). Polyposis and early cancer in a patient with low penetrant mutations in MSH6 and APC: Hereditary colorectal cancer as a polygenic trait. Int. J. Colorectal Dis..

[53] Jasperson K.W., Samowitz W.S., Burt R.W. (2011). Constitutional mismatch repair-deficiency syndrome presenting as colonic adenomatous polyposis: Clues from the skin. Clin. Genet..

[54] Plaschke J., Linnebacher M., Kloor M., Gebert J., Cremer F.W., Tinschert S., Aust D.E., von Knebel Doeberitz M., Schackert H.K. (2006). Compound heterozygosity for two MSH6 mutations in a patient with early onset of HNPCC-associated cancers, but without hematological malignancy and brain tumor. Eur. J. Hum. Genet..

[55] Wimmer K., Kratz C.P., Vasen H.F., Caron O., Colas C., Entz-Werle N., Gerdes A.M., Goldberg Y., Ilencikova D., Muleris M. (2014). Diagnostic criteria for constitutional mismatch repair deficiency syndrome: Suggestions of the European consortium ‘care for CMMRD’ (C4CMMRD). J. Med. Genet..

[56] Gallon R., Phelps R., Hayes C., Brugieres L., Guerrini-Rousseau L., Colas C., Muleris M., Ryan N.A.J., Evans D.G., Grice H. (2023). Constitutional Microsatellite Instability, Genotype, and Phenotype Correlations in Constitutional Mismatch Repair Deficiency. Gastroenterology.

[57] Gallon R., Brekelmans C., Martin M., Bours V., Schamschula E., Amberger A., Muleris M., Colas C., Dekervel J., De Hertogh G. (2024). Constitutional mismatch repair deficiency mimicking Lynch syndrome is associated with hypomorphic mismatch repair gene variants. npj Precis. Oncol..

[58] Szabo E., Blackburn E., Amodeo O.N., Nadeau S., Radecki A.A., Grady J.P., Rath A., Heinen C.D. (2025). A Cell-Based Functional Assay Calibrated for Analysis of MSH6 and MSH2 Mismatch Repair Gene Variants. Hum. Mutat..

[59] Li L., Hamel N., Baker K., McGuffin M.J., Couillard M., Gologan A., Marcus V.A., Chodirker B., Chudley A., Stefanovici C. (2015). A homozygous PMS2 founder mutation with an attenuated constitutional mismatch repair deficiency phenotype. J. Med. Genet..

[60] Bougeard G., Olivier-Faivre L., Baert-Desurmont S., Tinat J., Martin C., Bouvignies E., Vasseur S., Huet F., Couillault G., Vabres P. (2014). Diversity of the clinical presentation of the MMR gene biallelic mutations. Fam. Cancer.

[61] Lavoine N., Colas C., Muleris M., Bodo S., Duval A., Entz-Werle N., Coulet F., Cabaret O., Andreiuolo F., Charpy C. (2015). Constitutional mismatch repair deficiency syndrome: Clinical description in a French cohort. J. Med. Genet..

[62] Garrett A., Allen S., Loong L., Durkie M., Drummond J., Burghel G.J., Robinson R., Callaway A., Field J., Frugtniet B. (2026). CanVIG-UK Consensus Specification for Cancer Susceptibility Genes of ACGS Best Practice Guidelines for Variant Classification, Version 3.21.

[63] Nykamp K., Anderson M., Powers M., Garcia J., Herrera B., Ho Y.Y., Kobayashi Y., Patil N., Thusberg J., Westbrook M. (2017). Sherloc: A comprehensive refinement of the ACMG-AMP variant classification criteria. Genet. Med..

[64] Houge G., Bratland E., Aukrust I., Tveten K., Zukauskaite G., Sansovic I., Brea-Fernandez A.J., Mayer K., Paakkola T., McKenna C. (2024). Comparison of the ABC and ACMG systems for variant classification. Eur. J. Hum. Genet..

[65] Houge G., Laner A., Cirak S., de Leeuw N., Scheffer H., Dunnen J.T.D. (2022). Stepwise ABC system for classification of any type of genetic variant. Eur. J. Hum. Genet..

