# Supplementary material for: The Tyrolean Founder MLH1 Variant c.836T>G Causes Lynch Syndrome Due to a Leaky Splice Effect
Source: Biomolecules. 2026 Aug 17;16(8):1200. doi: 10.3390/biom16081200 (PMC13510572; doi:10.3390/biom16081200)
Supplement: Supplementary file 1 [file biomolecules-16-01200-s001.zip › biomolecules-4444793-Supplementary Figures.pdf]

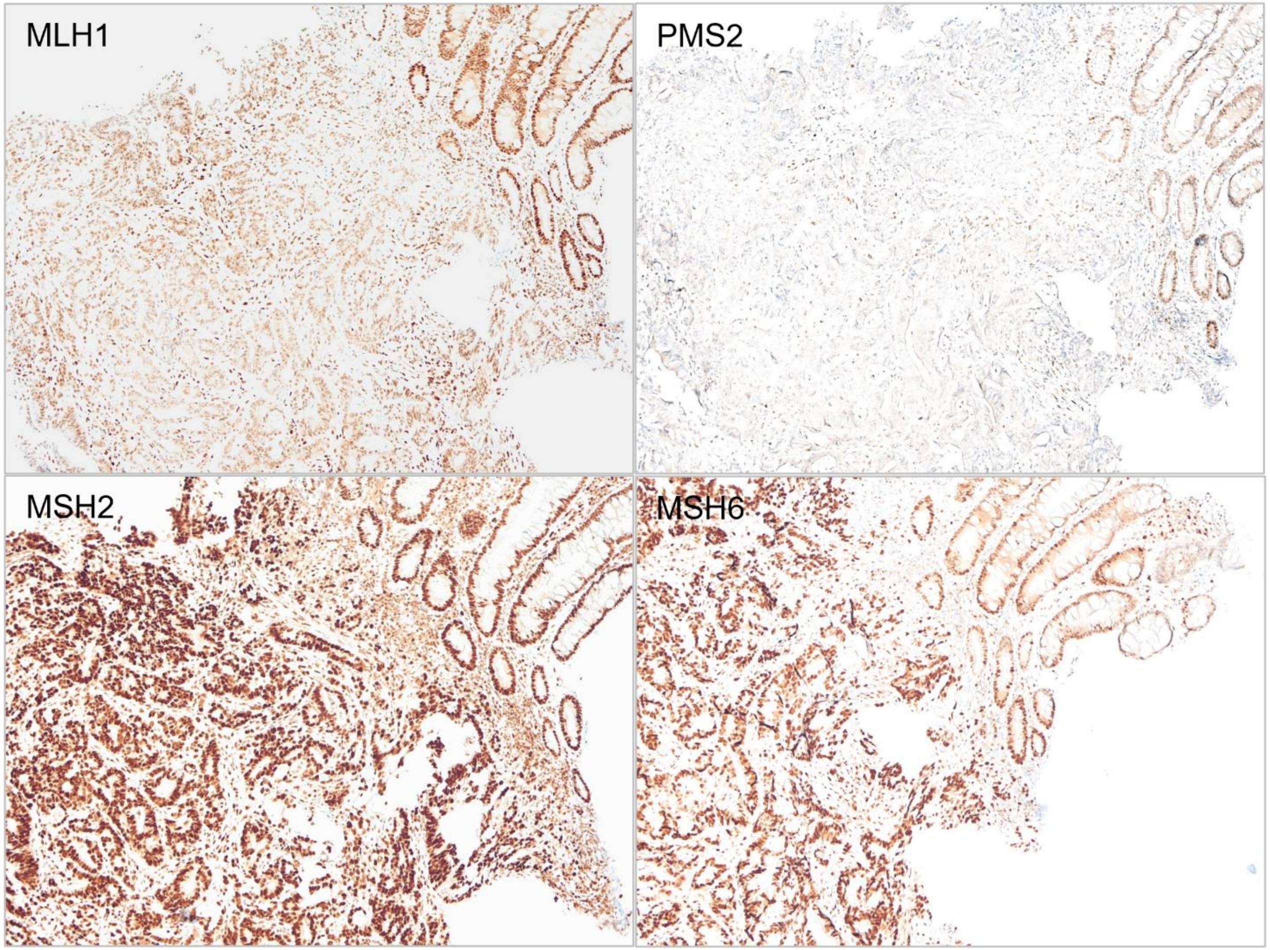

Supplementary Figure S1. Immunohistochemical (IHC) staining of the four mismatch repair (MMR) proteins in colorectal cancer tissue from patient P3 shows PMS2 expression loss with intact expression of the other MMR proteins.

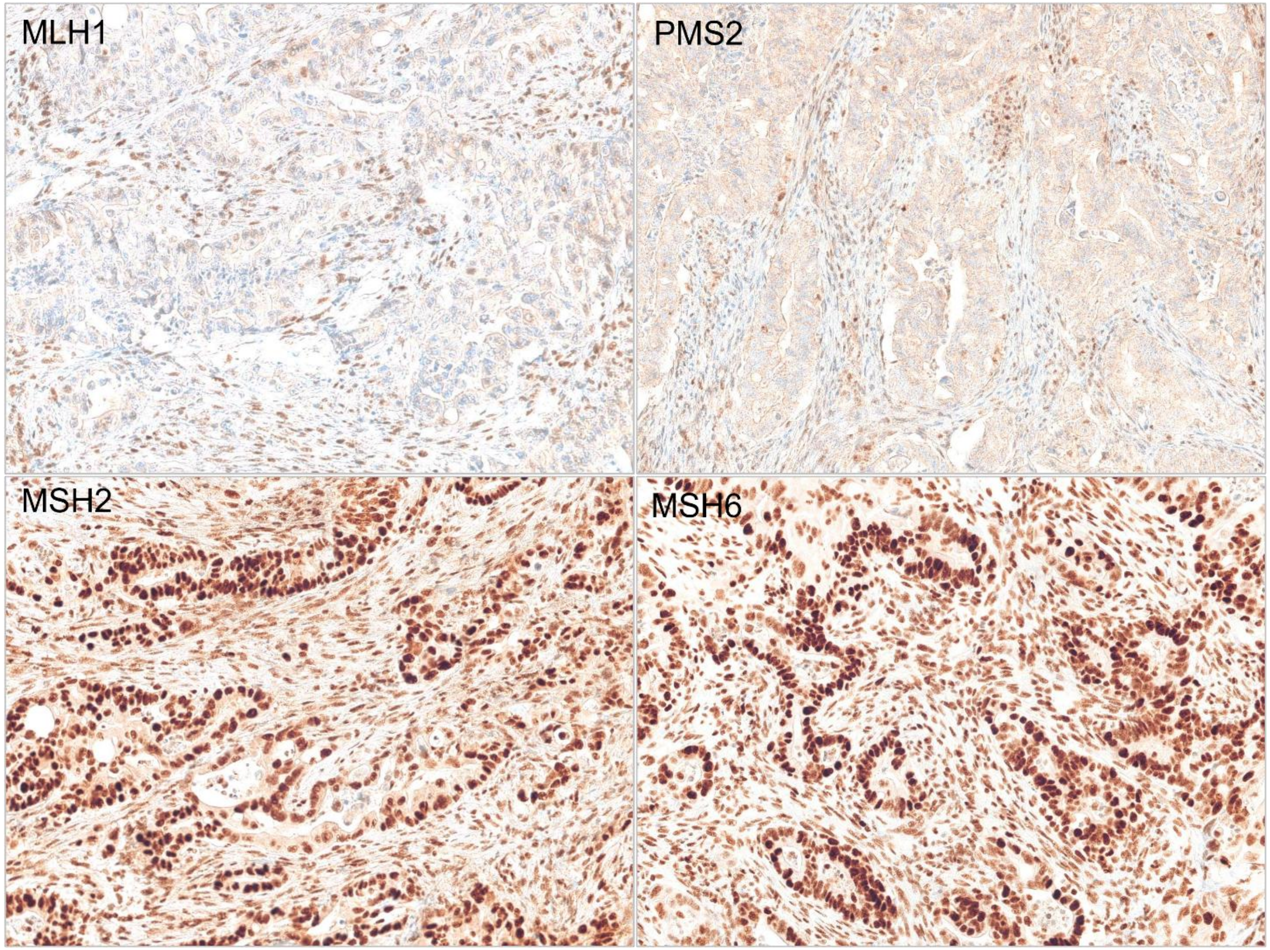

Supplementary Figure S2. Immunohistochemical (IHC) staining of the four MMR proteins in small bowel (intestine) cancer of patient P6 shows MLH1 and PMS2 expression loss with intact expression of the proteins MSH2 and MSH6.

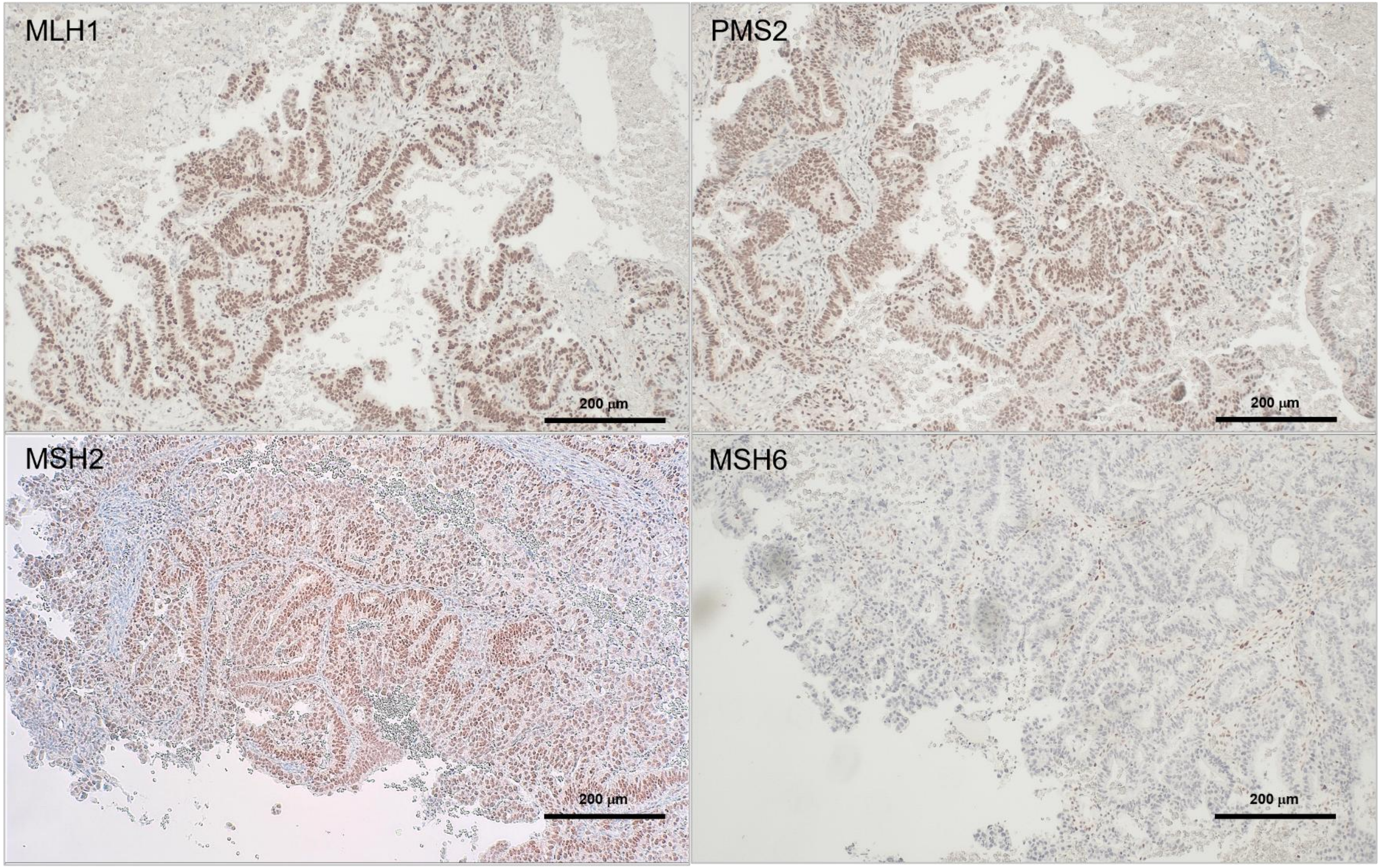

Supplementary Figure S3. Immunohistochemical (IHC) staining of the four MMR proteins in endometrial cancer tissue of patient P4 shows MSH6 expression loss with intact expression of the other MMR proteins.

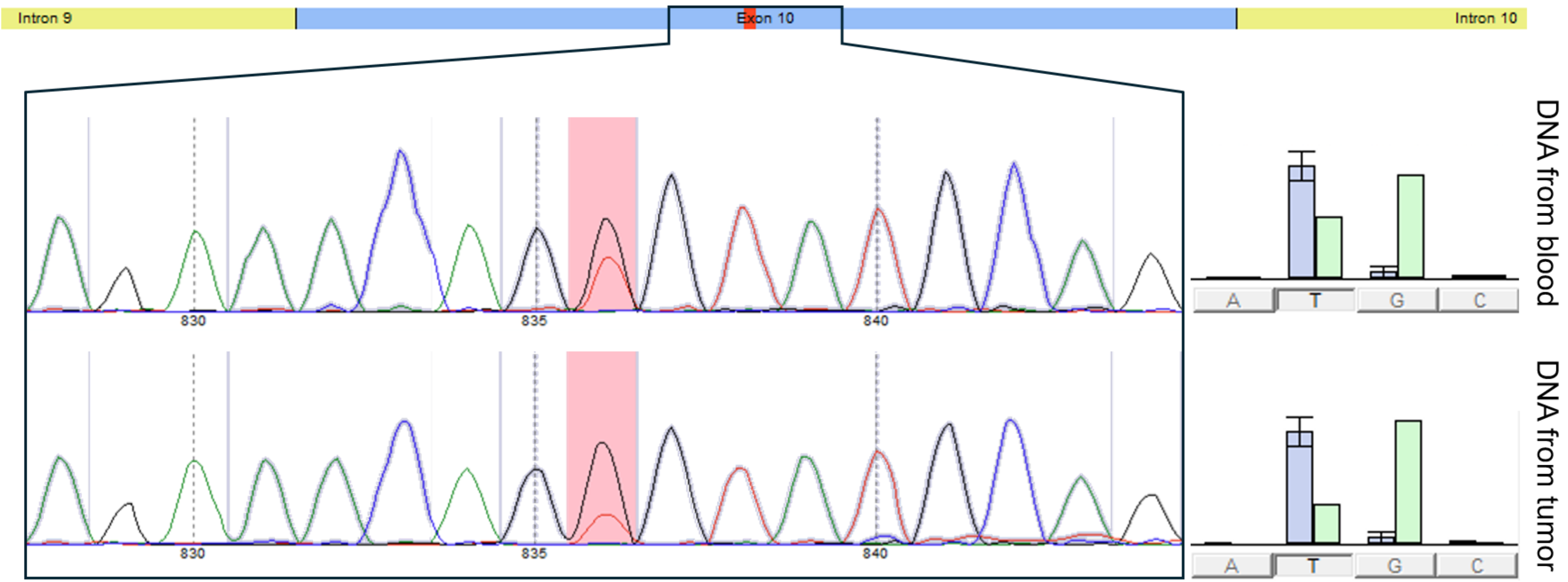

Supplementary Figure S4. Sanger sequencing of the *MLH1* variant c.836T>G and flanking nucleotides in DNA from blood (upper panel) and tumor DNA (lower panel) from patient P1

The sequenced region is shown schematically above the electropherograms which show nucleotides A, T, C, and G as green, red, blue, and black peaks, respectively. The position in the *MLH1*-transcript is shown below each electropherogram and the variant c.836T>G is highlighted with pink underlay. The area under the peak of the wild-type T and the variant G at position c.836 is shown in green bars for the patient's blood and tumor as well as for 69 controls (blue bar, incl. standard deviations). Note that the wild-type T is reduced more than 50% in the tumor DNA indicating LOH. NGS sequencing of tumor DNA confirmed the reduction of the wild-type T-allele to 27% of the reads while the variant G at position c.836 was present in 73% of the reads (data not shown).

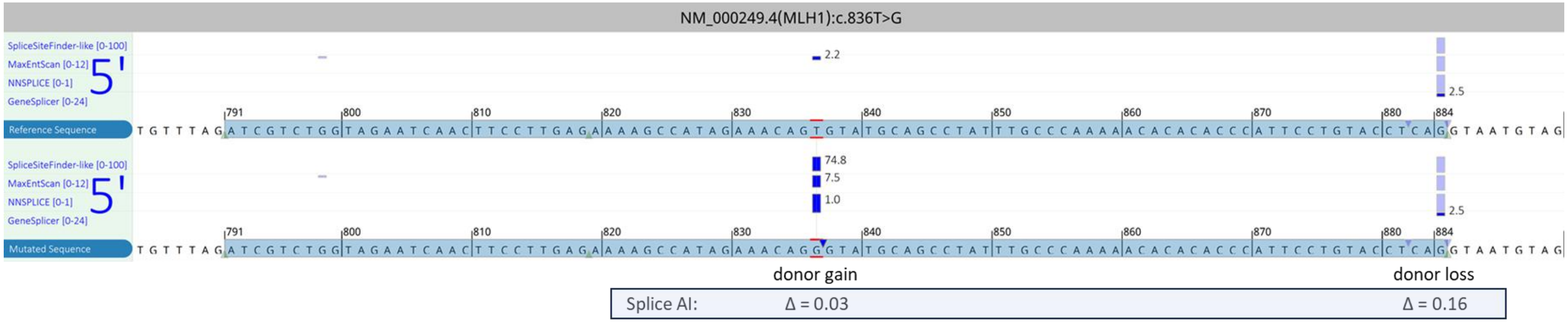

Supplementary Figure S5. Graphical representation of the predicted splice effect of *MLH1* variant c.836T>G

Screen shots taken from Alamut Visual Plus v. 1.8.1 by SOPHiA GENETICS software show the wild-type sequence (top) and variant sequence (bottom) of *MLH1* exon 10 with light blue background, while the flanking intronic sequences have no background color. The wildtype T at position c.836 and the variant G are highlighted in red. The 5'-splice site scores predicted by the programs SpliceSiteFinder-like, MaxEntScan, NNSPLICE and GeneSplicer are shown as vertical bars with the bar size corresponding to the score. The splice site scores with differences between the wild-type and variant sequence are highlighted by dark blue bars whereas unchanged scores are represented by light blue bars. The Δ scores calculated by SpliceAI for the predicted de novo 5'-splice site (donor gain) and the natural 5'-splice site of exon 10 (donor loss) are shown below these screen shots.

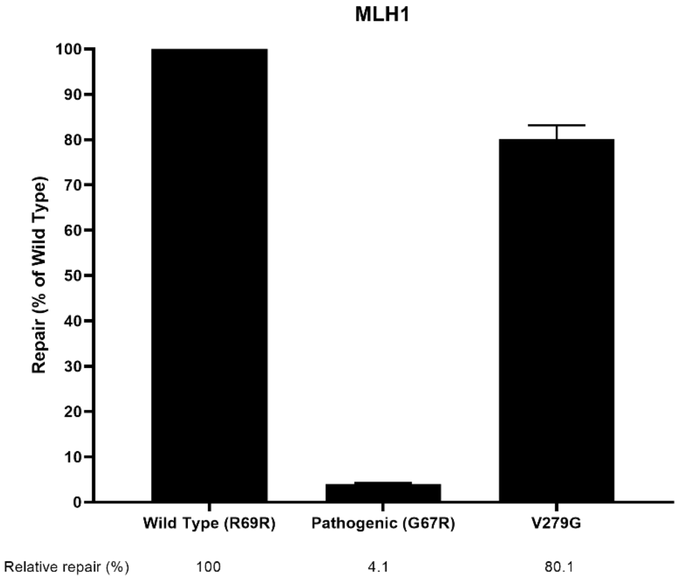

Supplementary Figure S6. Relative repair efficiency of MLH1 missense variant p.Val279Gly

The CIMRA assay demonstrates that the MLH1 missense variant p.Val279Gly (V279G) has a relative repair capacity of 80.1%, while the known pathogenic missense variant p.Gly67Arg (G67R) used as control has only 4.1% of repair capacity of the wild-type (100%).

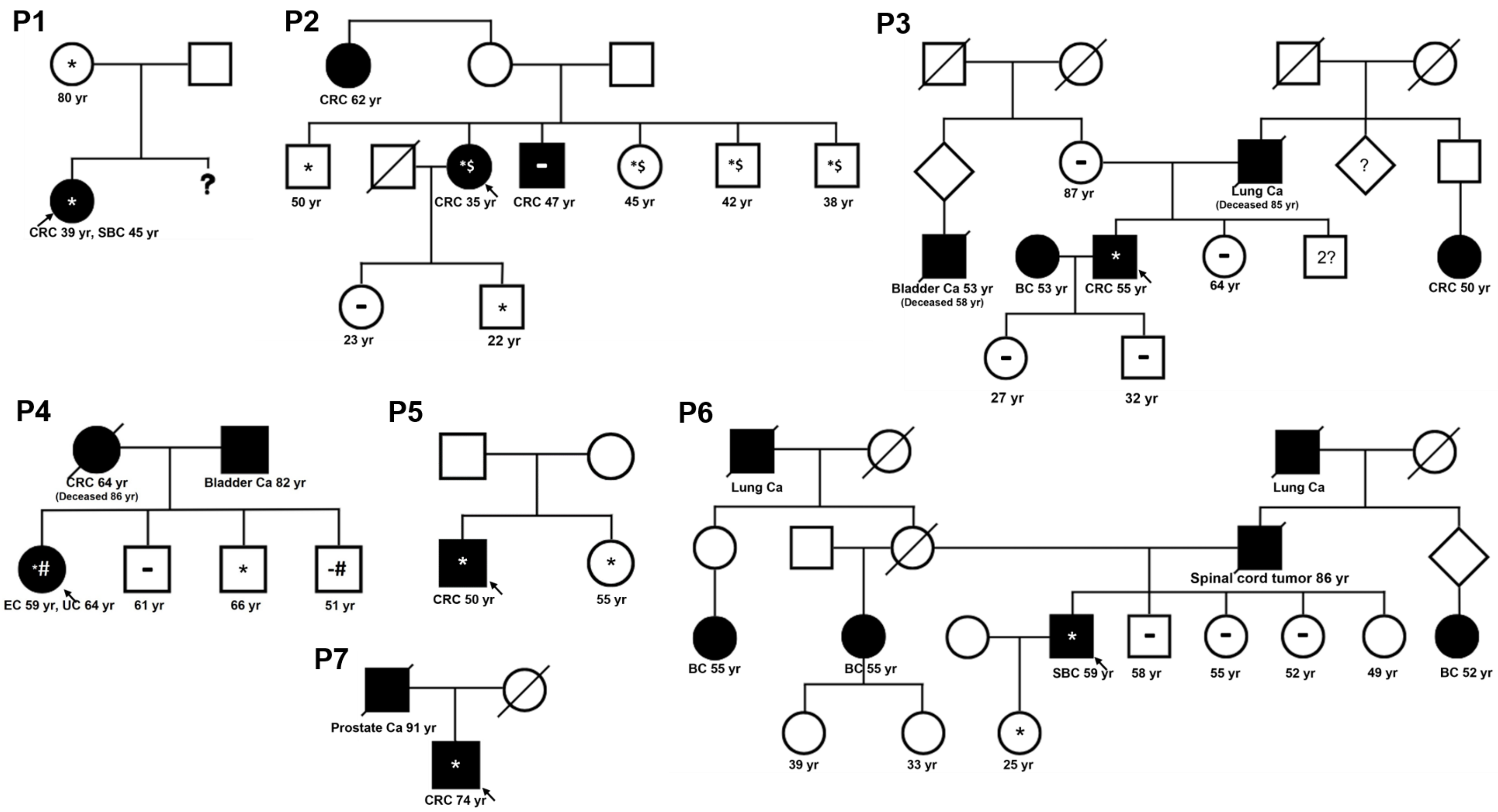

Supplementary Figure S7. Pedigree of seven probands

Probands are indicated with an arrow. For all probands and family members with cancer (filled symbols) the age of cancer onset is given. For all tested individuals without cancer (symbols without filling) the age at predictive testing is given. * indicates a carriers of *MLH1* variant c.836T>G; family members who tested negative for *MLH1* c.836T>G are indicated with - ; [$] indicates carriers of the *MSH6* variant of unknown significance (VUS) c.2561_2563delAGA, p.(Lys854del), identified in addition to c.836T>G in patient P2; [#] indicates carriers of the *MSH6* pathogenic variant c.3103C>T, p.(Arg1005Ter), identified in P4. Abbreviations: Ca, cancer; BC, breast cancer; CRC, colorectal cancer; EC, endometrial cancer; SBC, small bowel (intestine) cancer; UC, urothelial cancer; yr, years.
